# Carbon fiber based electrochemical sensor for sweat cortisol measurement

**DOI:** 10.1038/s41598-018-37243-w

**Published:** 2019-01-23

**Authors:** M. Sekar, M. Pandiaraj, S. Bhansali, N. Ponpandian, C. Viswanathan

**Affiliations:** 1 Department of Nanoscience & Technology, Bharathiar University, Coimbatore, Tamil Nadu India; 2 Electrodics and Electrocatalysis Division, CSIR-Central Electrochemical Research Institute, Karaikudi, Tamil Nadu India; 3 Bio-MEMS and Microsystems Laboratory, Department of Electrical and Computer Engineering, Florida International University, Miami, FL USA

## Abstract

This study examines the use of a conductive carbon fiber to construct a flexible biosensing platform for monitoring biomarkers in sweat. Cortisol was chosen as a model analyte. Functionalization of the conductive carbon yarn (CCY) with ellipsoidal Fe_2_O_3_ has been performed to immobilize the antibodies specific to cortisol. 1-Ethyl-3-(3-dimethylaminopropyl) carbodiimide (EDC) and N-Hydroxysuccinimide (NHS) chemistry has been used to immobilize the antibodies onto the Fe_2_O_3_ modified CCY. Crystallinity, structure, morphology, flexibility, surface area, and elemental analysis were studied using X-ray diffraction (XRD), Fourier transform infrared spectroscopy (FT-IR), Raman spectroscopy, Field emission scanning electron microscopy with energy dispersive X-ray spectroscopy (FE-SEM/EDS) and Brunauer–Emmett–Teller (BET) analysis. Mechanical properties of the fiber such as tensile strength, young’s modulus have also been investigated. Under optimal parameters, the fabric sensor exhibited a good linearity (r^2^ = 0.998) for wide a linear range from 1 fg to 1 μg with a detection limit of 0.005 fg/mL for the sensitive detection of cortisol. Repeatability, reliability, reproducibility, and anti-interference properties of the current sensor have been investigated. Detection of cortisol levels in human sweat samples has also been investigated and the results were validated with commercial chemiluminescence immunoassay (CLIA) method.

## Introduction

Analysis of biomarker levels in sweat is increasingly important as it would give real time information about human performance, health and wellbeing. The World Health Organization (WHO) lists cardiovascular disease (CVD) as one of the leading cause of death globally and the mortality rate due to CVD has increased annually than any other cause in the developed and developing countries^1^. CVDs can be caused by a range of factors and disorders including high blood pressure, cholesterol, diabetes, obesity and overweight, smoking and stress^2^. Among these factors, psychosocial stress is reported to be major cause for CVD^3^. Suffering from stress is becoming a global issue and it affects the peoples’ ability to take decision at work including police, soldiers in combat, athletes or anybody in emergency situation. There is growing evidence that stress is harmful to health as it alters the immune system causing increased propensity to infections.

Non-invasive point-of-care (POC) monitoring devices will be helpful in such scenarios as they would aid rapid diagnosis and treatment of people suffering from stress^4,5^. Another added advantage of the POC devices is shifting of theronosis from hospital to home based supervision for accessing human performance. Cortisol, called as the stress hormone, has been recognized as a key element in the psychobiology of the stress response and related negative health outcomes. It regulates various physiological processes and plays an important role in homeostasis of the cardiovascular, immune, renal, skeletal and endocrine system and regulating blood pressure. Insufficient amounts of cortisol can cause nonspecific symptoms such as weight loss, low blood pressure, fatigue, muscle weakness and abdominal pain. Too much cortisol can cause increased blood pressure, high blood sugar, obesity, fragile skin, purple streaks on the abdomen, muscle weakness and osteoporosis^6–10^. Secreted cortisol finds its way into the circulatory system and can be found in detectable quantities in several bio-fluids in human body. When compared to various bio-fluids, sweat is the most extensively evaluated non-invasive body fluid as it contains plethora of medical information for diagnosis. It is comparatively easier to stimulate, collect and analyze^11,12^. Sweat based monitoring overcomes many of the shortcomings related with blood based assays.

Methods used to detect cortisol includes, fluorescence assay calorimetric^13^, High performance liquid chromatography (HPLC)^14^, reverse phase chromatography^15^, enzyme linked immunosorbent assays (ELISA)^16,17^, competitive protein-binding assays^18,19^ and surface plasmon resonance biosensor (SPR)^20^. But they suffer from long detection time, lengthy sample preparation time and analysis, low sensitivity and cost. Recently, novel electrochemical immunosensor, aptamer sensor and imprinted sensor^21,22^ method has been developed to detect cortisol owing to their fast analysis speed, high sensitivity, and low cost^7,23,24^. However, these sensors involve the use of traditional Au metallic disk, glassy carbon electrode (GCE), metal electrodes, metallic fibers or printed electrodes. Fabrication cost, self-oxidation and internal resistance are common in these electrodes and it easily influences the electrochemical activity of the sensor. Therefore, there is increasing demands for developing cost-effective electrode materials capable of providing reliable sensing performance is much needed^23,25,26^.

In recent years, there has been growing interest in the development of wearable biosensors, which would open up a revolutionary opportunity to monitor patients even in remote areas which is key for the development of modern healthcare^27^. To meet the special requirement of wearable sensor, the binder-free material with free-standing structure emerges which can effectively simplify the electrode preparation process and improve the electrochemical performance.

Carbon yarn, an outstanding class of carbon materials, with carbon atoms bonded together in crystals that are aligned parallel to the long axis of the fiber. The crystal alignment gives the fiber high strength-to-volume ratio. Fiber materials have been widely applied in the field of electrochemistry and composite materials due to its intrinsic carrier mobility, electrical conductivity, environmental stability, superior mechanical properties, low weight and high temperature tolerance as well as potential for production at low cost^28–31^. Fabrics based on conductive fibers represent an excellent class of substrates for developing wearable sensors because they would be in constant contact with the skin^32,33^. On the other hand, among the semiconductor nanomaterials, Hematite (α- Fe_2_O_3_), which is the most stable iron oxide under ambient conditions with n-type semiconducting properties has been investigated extensively for electrochemical sensing, catalysis, solar cell and energy conversion applications due to its abundance, low cost, high stability and environmental benignity with great scientific and technical importance^34,35^. Therefore, a novel design of the structure with Fe_2_O_3_ nanostructure coated on flexible carbon yarn substrate is highly desirable to achieve the flexible binder free electrode with high electrochemically sensing performance^36^.

Recently, our group developed new approach for SnO_2_ coating on carbon yarn and it was directly used as binder free electrode for the sensitive detection of dopamine (DA)^31^. Also, Mahesh K.P.O. *et al*., reported that the porous Fe_2_O_3_ nanoparticles are directly grown on carbon cloth using a simple hydrothermal method for employment as a flexible and wearable electrochemical electrode for the detection of DA^36^. A flexible strain-gauge sensor for monitoring heart rate was developed by Yeon Hwa Kwak *et al*.^37^. It demonstrated that the nanofibers can be used as an alternative active sensing element for wearable gas sensor application. Recently, polyaniline (PANI)/polyacrylonitrile (PAN) nanofibers developed by electro spinning method exhibited a high sensitivity and fast response towards ammonia (NH_3_) detection^38^. Similarly, a new method to the real time analysis of sodium and chloride in human sweat using wearable fibers based biosensors has been reported recently^39^. Moreover, P. Manickam *et al*., described the construction of biochemical sensor platform on thread for continuous monitoring of cortisol levels. The TiO_2_ anchored carbon yarn used to detect cortisol with improved sensitivity and LOD of 10 pM^40^.

Generally, the nanostructure of Fe_2_O_3_ such as nanoparticles, nanosphere, nanotubes, nanosheets and nanoellipsoid with the high surface area can shorten the transport path of electrons and ions. Also, these morphology changes of the two types of electrodes may affect the dimensions of the reactive regions and thus alter the electrochemical response. Most of these functions depend strongly on the structure and morphology of the semiconductor materials, and one-dimensional (1D) α-Fe_2_O_3_ nanostructured materials that exhibits better electrochemical performance.

With these motivations, we have prepared ellipsoid Fe_2_O_3_ on CCY flexible electrode platform for developing electrochemical immunosensors for monitoring cortisol levels. Anti-C_mab_ were immobilized onto the Fe_2_O_3_/CCY electrode using EDC/NHS chemistry. The fabricated immunosensor was tested for cortisol level in human sweat samples and validated with commercial CLIA.

## Materials and Methods

### Chemical and reagents

Iron (III) chloride Hexahydrate, Pluronic f127, 1-Ethyl-3-(3-dimethylaminopropyl) carbodiimide (EDC) and N-Hydroxysuccinimide (NHS), Bovine Serum Albumin (BSA), Hydrocortisone (Cortisol), Progesterone,  Cholesterol, Cortisone was purchased from Sigma Aldrich. The monoclonal cortisol antibodies (Anti-cortisol, Anti-C_m__ab_) (2330–4809) were purchased from EastCoast Bio. The working solutions of cortisol were prepared by dilution in phosphate buffered saline (PBS) (10 mM, pH 7.0). All the chemicals were of analytical grade and were used without further purification.

Bleached and scoured CCY with the density and diameter of 0.35 g/cm^3^ and ~350 μm was purchased from Vinpro Tech, Hyderabad, India. The purchased CCY was ultrasonicated and dried at ambient temperature under vacuum for 24 h to remove any impurities prior to characterization and material deposition.

### Synthesis of ellipsoidal Fe_2_O_3_ nanostructures integration with CCY (Fe_2_O_3_/CCY)

Ellipsoidal Fe_2_O_3_ was integrated with CCY using the simple hydrothermal method, wherein stoichiometric amounts of pluronic f127 were added to an aqueous solution of FeCl_3_.6H_2_O with continuing magnetic stirring. Following this, the obtained homogeneous solution was transferred into a Teflon vial (65 mL). CCY was immersed in this solution, placed in a sealed autoclave and heated at 180 °C for twelve hours. The resulting yarn was allowed to cool down to room temperature naturally, the yarn was collected and washed with alcohol, double distilled water for four times to remove remaining impurities. Then the product was dried at 80 °C overnight.

### Immobilization of Anti-C_mab_ onto Fe_2_O_3_/CCY electrode

**Figure 1:**
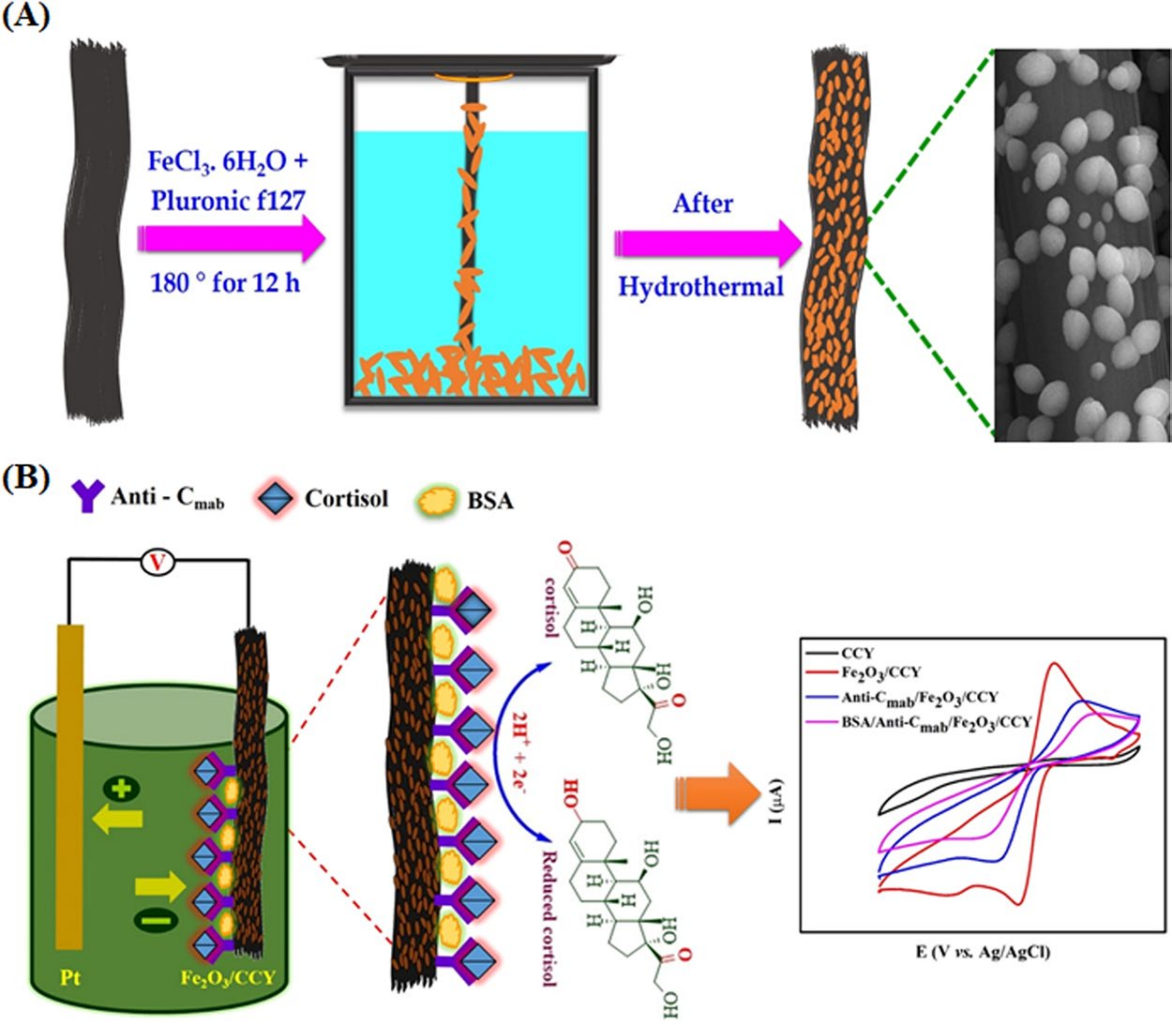
(**A**) Schematic for Fe_2_O_3_/CCY preparation by hydrothermal method; (**B**) immobilization Anti-C_mab_ onto Fe_2_O_3_/CCY to fabricate cortisol immunosensor.

Fe_2_O_3_ coated CCY was directly used as the mediator or conductive additive materials free working electrode for the electrochemical detection of cortisol. The working electrode was kept at ~5 cm in length and 4 cm length fiber was soaked into the electrolyte solution. A standard process, reported previously, has been utilized to immobilize Anti-C_mab_ covalently onto Fe_2_O_3_/CCY electrode^41^. The immobilization of Anti-C_mab_ with Fe_2_O_3_/CCY electrode was achieved via electrostatic interaction using EDC as the coupling agent and NHS as the activator due to the high isoelectric point (IEP) of Fe_2_O_3_ (8.5) and antibody (4.5) using physical adsorption. To fabricate the electrochemical immunosensor electrode, 70 μL of 2 μg/mL, Anti-C_mab_ solution was mixed with the Fe_2_O_3_/CCY in 5 mL PBS (10 mM, pH 7.0) solution containing 0.4 M NHS and 0.4 M EDC and incubated for 2 h in a humid chamber. The fabricated Anti-C_mab_/Fe_2_O_3_/CCY electrode was washed with PBS (10 mM, pH 7.0) to remove any loosely bound molecules. Following this the sensor electrode was immersed in 50 μL of BSA (10 μg/mL) in PBS (10 mM, pH 7.0) and incubated for 30 mins for blocking nonspecific binding sites on the Anti-C_mab_/Fe_2_O_3_/CCY electrode surface. As fabricated BSA/Anti-C_mab_/Fe_2_O_3_/CCY electrode was further washed using PBS (10 mM, pH 7.0) and stored at 4 °C. Figure 1a,b shows the schematic of Fe_2_O_3_/CCY preparation by hydrothermal method and subsequent sensor fabrication.

### Characterization

Structural features of Fe_2_O_3_ nanostructures were investigated using a PANalytical (X’Pert-Pro) diffractometer (using Cu Kα radiation at a wavelength of 1.5406 Å) and the XRD patterns were recorded in the diffraction angle (2 Theta) ranging from 20° to 70°. The functional groups of the nanocomposite materials were identified by Bruker Tensor 27 Fourier transform infrared spectrometer (FT-IR). Raman spectra were recorded at using a Horiba Jobin-LabRam-HR system at 514 nm excitation focused through a 100x microscope objective for a total spot size of 1 μm. Excitation power was held constant at 150 μW for all samples. The morphologies were observed by field emission scanning electron microscopy (FESEM) on FEI Quanta-250 FEG microscope. The compositions of the materials were confirmed using energy dispersive X-ray spectrometry (EDS) by the FESEM attachment. The high-resolution transmission electron microscopy (HRTEM) images were recorded on a JEM-2100FS instrument (JEOL) operating at 200 kV to further confirm the morphology of the samples. Surface area measurements were carried out using Brunauer-Emmett-Teller (BET) Quantachrome Nova 1200e (USA) instrument with N_2_ as the analysis gas. The mechanical properties of the pristine and modified CCY were examined using the universal testing machine (UTM) (Zwick Roell).

### Electrochemical characterizations

Electrochemical measurements were carried out to analyze the electro active behavior of the electrodes. The electro activity of CCY, Fe_2_O_3_/CCY, Anti-C_mab_/Fe_2_O_3_/CCY and BSA/Anti-C_mab_/Fe_2_O_3_/CCY electrodes was studied in a 20 mL of PBS (pH 7.0) containing 5 mM Fe(CN)_6_^3−/4−^. The conventional three-electrode electrochemical workstation (BioLogic SP-50) was used for all the experiment. In the cell, the BSA/Anti-C_mab_/Fe_2_O_3_/CCY was used as the working electrode. A platinum wire and Ag/AgCl was used as the counter and reference electrodes, respectively. All the electrochemical measurements were made thrice and the average is used for analysis.

### Sweat collections for analysis

Sweat samples were collected immediately after 30–45 minutes of vigorous exercise were the age group of 27–35 with proper permission. The exercise entailed either running on treadmill or rowing on an ergometer for a minimum of 10 minutes. Sweat samples were collected in the evening to examine whether sweat cortisol concentrations approximated the well-established theoretical value. For sweat collection, a cotton swab was rubbed over the scalp hair and neck, allowed to saturate and placed it in a 5 mL Eppendorf tube^42^. The collected samples were centrifuged for 5 minutes and 1 mL of the supernatant was pipetted out in and stored at −20 °C to maintain its biological characteristics. These samples were further used to detect cortisol using electrochemical immunosensor and for comparison with commercial cortisol assay. Ethical approval for this study was granted by the Department Ethics Committee of Bharathiar University (BU). All experiments were performed in accordance with relevant guidelines and regulations and all experimental protocols were approved by the Department Ethics Committee, BU. Informed consent was obtained from all subjects.

## Results and Discussion

### Structure and morphology of the CCY and α-Fe_2_O_3_

**Figure 2:**
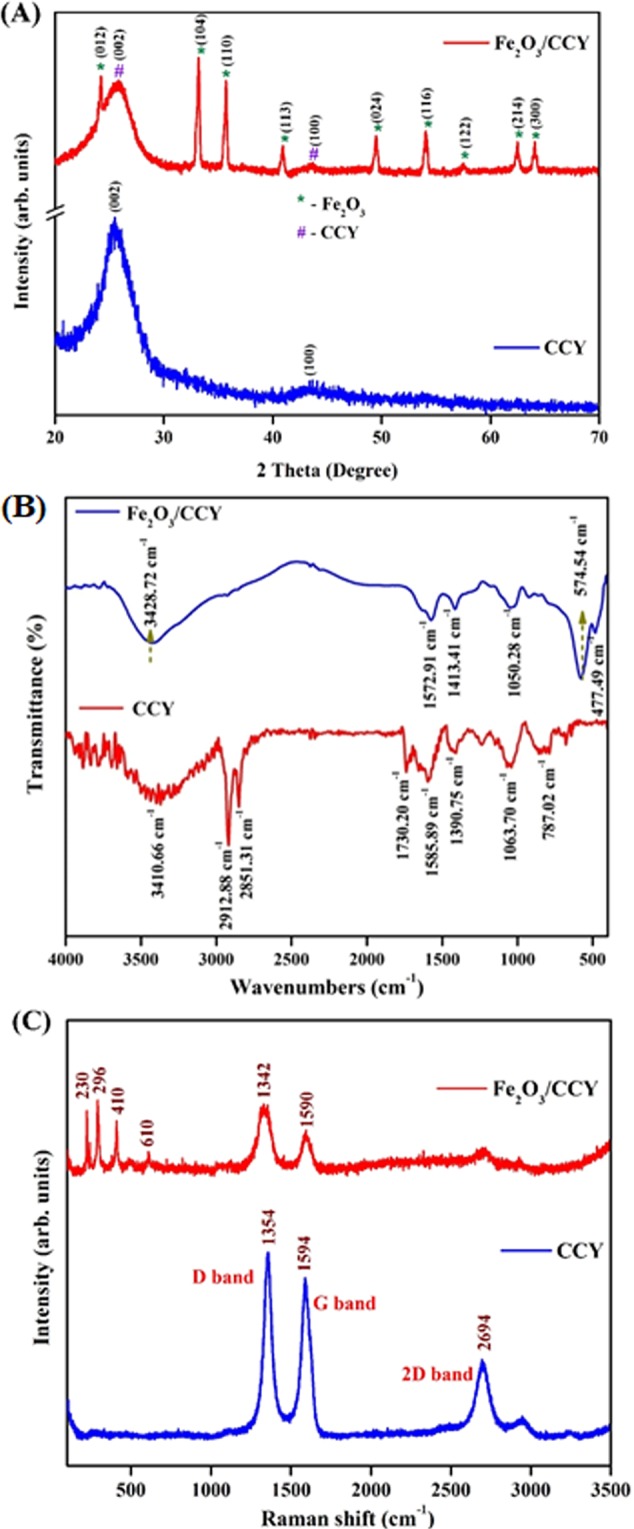
(**A**) X-ray diffraction patterns (**B**) FT-IR spectrum and (**C**) Raman spectra of CCY and Fe_2_O_3_/CCY.

The crystal structures and the phases of samples were investigated by XRD measurements and the resulting XRD diffraction patterns are displayed in Fig. 2A. The bare CCY shows only two broad diffraction peaks at 25.8° and 43.6° which could be indexed to the (002) and (100) planes of amorphous carbon, respectively^43,44^. For Fe_2_O_3_/CCY, the signature patterns for carbon yarn at 25.9° (002) and several new diffraction peaks at 24.2°, 33.2° (major), 35.86°, 40.96° and 54.13°corresponding to (012), (104), (110), (113) and (116) planes, respectively were observed which are consistent with the standard XRD patterns of rhombohedral α-Fe_2_O_3_ (JCPDS No. 84-0309). This results prove the successful formation of Fe_2_O_3_ on CCY with high purity^45^.

In order to investigate the interaction between Fe_2_O_3_ and CCY, the FT-IR spectrum has been performed. Figure 2B shows the chemical information and major functional groups existing in the pure CCY and Fe_2_O_3_/CCY obtained using FT-IR. For pristine CCY, a characteristic peak at 1585 cm^−1^ observed for C=C stretching and the band at about 1063 cm^−1^ is consigned to C–C stretching vibration in the carbon. The peaks at 781 cm^−1^, 2851 and 2912 cm^−1^ were ascribed to the bending and stretching vibrations of the C-H bond. A strong absorption band at 3410 cm^−1^ corresponds to O-H stretching vibration along with O-H bending and deformation vibration at 1730 and 1390 cm^−1^ respectively^46^. The absorption bands at 477 and 574 cm^−1^ are attributed to the Fe-O-Fe and Fe-OH bond vibration, respectively present in Fe_2_O_3_ integrated CCY. The bands at 1572 and 3428 cm^−1^ are assigned to bending modes of O-H^47,48^. In addition, weak peak around 1413 cm^−1^ roots in C-O deformation vibration, implying the existence of intramolecular hydrogen bonding between CCY and Fe-OH, which is beneficial in keeping the stability of material structure. A remarkable decrease in the absorption of C=O, O-H (deformation vibration) and the C-O groups were observed, proving that most of the oxygen containing groups were removed^31^. From the FT-IR results, we corroborate that the Fe_2_O_3_ are covered to the CCY surface uniformly.

Raman spectroscopy was used to further confirm the structural characteristics of nanocomposite electrode materials. Raman spectra of the pure CCY and Fe_2_O_3_/CCY are taken in the range of 100–3500 cm^−1^ and shown in Fig. 2C. The D-band located at 1355 cm^−1^ and the G-band at 1584 cm^−1^ are the characteristic Raman shifts of carbon, which can be observed in both pure CCY and Fe_2_O_3_/CCY. The G band assigned to the first order scattering of the E_2_g phonon of CCY represent the in plane bond stretching vibration of sp^2^-bonded carbon atoms in a 2D hexagonal lattice. The D band is associated with the breathing mode of K-point phonons of A_1_g symmetry with vibration of carbon atoms with angling bonds in plane terminations of disordered carbon yarn. In addition to this, the appearance of two weak peaks at 2693 and 2944 cm^−1^ can be assigned to the 2D band, which originates from the second-order Raman scattering process^49^. As we can see from the spectra of Fe_2_O_3_/CCY, the Fe_2_O_3_ sample exhibited the bands at 210, 274 388 and 610 cm^−1^ indicating the presence of Fe_2_O_3_ (hematite) phase with the D^6^_3d_ crystal space group^50,51^. As expected, the spectra of Fe_2_O_3_/CCY sample also exhibited peaks due corresponds to carbon. This result implies a tight integration of Fe_2_O_3_ nanostructures on CCY and supporting the XRD results very well.

**Figure 3:**
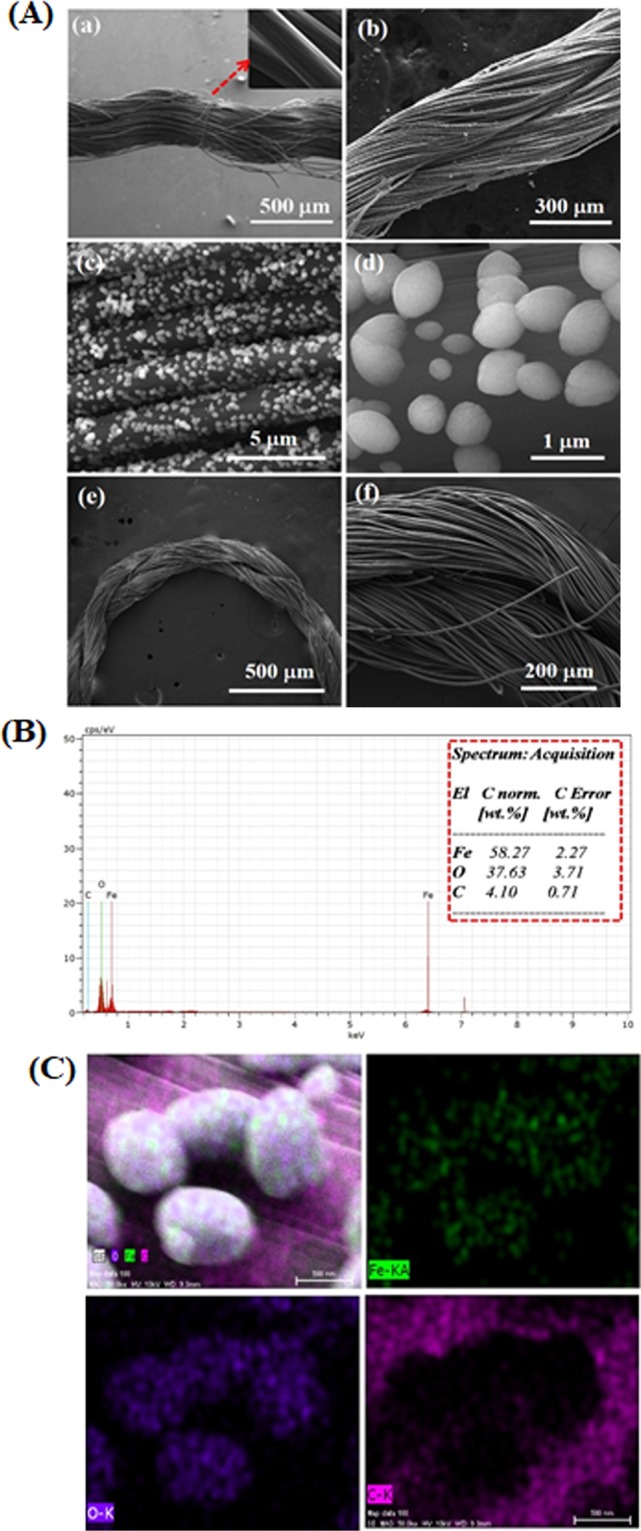
(**A**) FESEM images of (a) bare CCY, (b–d) Fe_2_O_3_/CCY and (e,f) Fe_2_O_3_/CCY electrode freely rolled up with tweezers; (**B**) EDS spectra Fe_2_O_3_/CCY; (**C**) corresponding FESEM image and EDS mapping of Fe_2_O_3_/CCY.

The morphology of the CCY and Fe_2_O_3_/CCY were elucidated by FESEM and the corresponding images are shown in Fig. 3A. As seen in Fig. 3A(a), the morphology of pure CCY is consists of smaller fibers and reveals that there are no impurities on the smooth surface. The inset figure shows the clearer version of the smooth fiber. High-magnification FESEM images shown in Fig. 3(b–d) provide clear information about the ellipsoidal Fe_2_O_3_ nanostructures. As depicted the carbon yarn surfaces are uniformly covered by ellipsoidal Fe_2_O_3_ nanostructures and it reveals that the material consists of uniform size with the diameter of 300–350 nm, while the length is ~800–850 nm. The large number of Fe_2_O_3_ nanoparticles were uniformly anchored onto the carbon yarn surface via self-assembly due to the differences in surface charges resulting in strong electrostatic interactions, which is expected to improve the electrochemical properties of Fe_2_O_3_ resulting in enhanced sensing performance. Figure 3A(e,f) show images of the integrated ellipsoidal Fe_2_O_3_/CCY electrode which can be freely rolled up with tweezers. It can be clearly observed that the electrodes exhibit excellent flexibility, which makes them possible for application in flexible and wearable devices.

The successful synthesis of Fe_2_O_3_ ellipsoid on CCY and its chemical composition was further confirmed by EDS mapping analysis. As shown in Fig. 3B, iron and oxygen are two elements coated on CCY apart from the carbon that relates to the substrate. Moreover, elemental mapping technique shown in Fig. 3C further established the presence of Fe_2_O_3_ on yarn. These images verify a homogeneous coating of the yarn with Fe_2_O_3_ nanoparticles.

**Figure 4:**
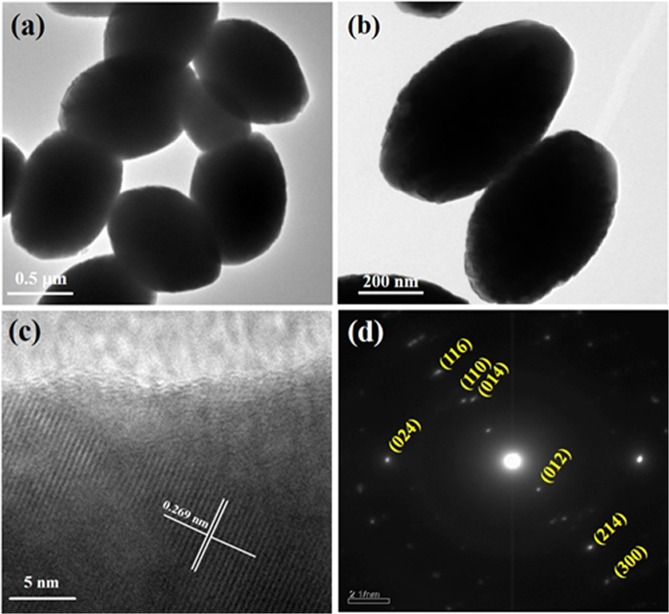
HRTEM images (**a**–**c**) and SAED pattern (**d**) of Fe_2_O_3_ nanoparticles.

To extensively study the morphology of the nanostructures, we have examined the HRTEM imaging and selected area electron diffraction (SAED) patterns for the synthesized Fe_2_O_3_. Figure 4(a,b) depicts the typical HRTEM images of Fe_2_O_3_ nanoparticles and indicates the ellipsoidal morphology with a homogeneously well dispersed structure which is consistent with the FESEM micrograph. The ellipsoidal Fe_2_O_3_ nanostructure is beneficial for electrode materials due to the large surface area. In Fig. 4c the uniform lattice structure without detectable defects and the calculated d spacing of 0.269 nm corresponds to the (104) planes of hematite could be clearly observed which can be correlated with XRD data. The corresponding SAED patterns are shown in Fig. 4d which indicates the polycrystalline nature of Fe_2_O_3_.

The BET surface area of the CCY and Fe_2_O_3_/CCY found to be 75.607 m^2^/g and 146.02 m^2^/g respectively. The uniform Fe_2_O_3_ nano-ellipsoids on CCY increases the surface area of the CCY nearly by two fold. Also, the mechanical properties of CCY and Fe_2_O_3_/CCY electrodes were investigated for its tensile, elongation and elastic modulus properties. The ultimate strength measured for Fe_2_O_3_/CCY is found to be 33.27 MPa and this value is higher than the pristine CCY (20.10 MPa). This indicates that the strength of the fiber is improved when it is modified with Fe_2_O_3_. Similarly, elongation and young’s modulus of the Fe_2_O_3_/CCY is 20.40% and 67.03 MPa were improved after Fe_2_O_3_ inclusion (7.53 % and 37.17 MPa).

### Electrochemical characterization

**Figure 5:**
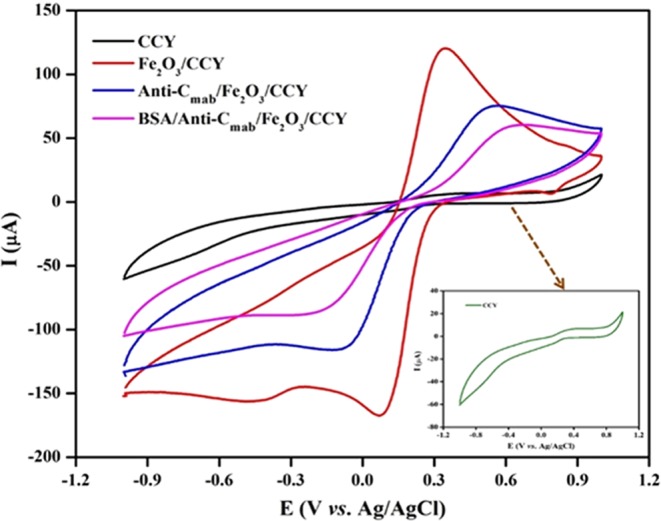
CV studies of step wise fabrication of BSA/Anti-C_mab_/Fe_2_O_3_/CCY immunoelectrode from CCY in PBS (10 mM, pH 7.0), [Insert: CV of bare CCY electrode].

The obtained Fe_2_O_3_/CCY was directly applied as a working electrode for electrochemical sensor to evaluate its cortisol sensing performance. CV was used to study the electro activity of the functionalized electrode to understand the electrochemical behavior of the electrode. Figure 5 shows the CV studies of the bare CCY, Fe_2_O_3_/CCY, Anti-C_mab_/Fe_2_O_3_/CCY, BSA/Anti-C_mab_/Fe_2_O_3_/CCY immunoelectrode in PBS (10 mM, pH 7.0). The bare CCY exhibited oxidation and reduction current magnitude in the range of ~10^−6^ A, which is a typical characteristic of bare CCY (as inset). The oxidation current response increases to 120.7 μA for Fe_2_O_3_/CCY electrode and it only shows the redox peak of Fe_2_O_3_ which_,_ the anodic oxidation peak corresponds to the oxidation of Fe^2+^ to Fe^3+^ and the cathodic peak corresponds to the reduction of Fe^3+^ to Fe^2+^. The magnitude current response decreased to 75.99 μA after the immobilization of Anti-C_mab_ onto Fe_2_O_3_/CCY electrode indicates the binding of Anti-C_mab_. The decreased current response is due to hindrance in electron charge resistance caused by the insulating nature of antibodies. Moreover, the electrochemical oxidation peak current response of BSA/Anti-C_mab_/Fe_2_O_3_/CCY immunoelectrode is observed to be lower than that of Anti-C_mab_/Fe_2_O_3_/CCY immunoelectrode. The decrement in the current response of BSA/Anti-C_mab_/Fe_2_O_3_/CCY immunoelectrode is revealing the hindrance in charge transfer due to the insulating behaviour of BSA via blocking of non-specific binding sites and suggests the successful immobilization of BSA onto the Anti-C_mab_/Fe_2_O_3_/CCY immunoelectrode.

### Effect of pH and scan rate

**Figure 6:**
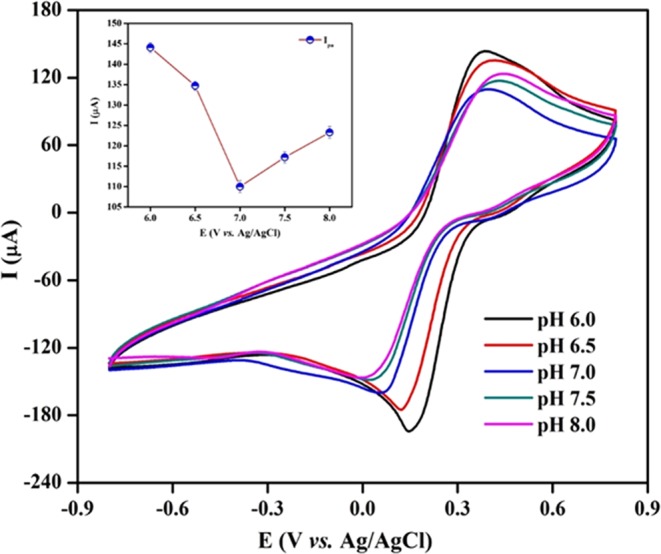
CV studies obtained for the BSA/Anti-C_mab_/Fe_2_O_3_/CCY immunoelectrode as a function of pH from 6.0 to 8.0. [Inset: plots of peak potentials versus pH values].

To investigate the optimal pH, the activity of BSA/Anti-C_mab_/Fe_2_O_3_/CCY immunoelectrode was investigated in the pH range of 6.0 to 8.0 (Fig. 6). It was observed that the oxidation and reduction peak current decreases on increasing pH from 6.0–7.0. Further increasing the pH up to 8.0, the peak current again linearly increased (as shown in insert of Fig. 6). However, it was observed the most stable oxidation and reduction peak area and current response is high for pH 7.0. Thus, pH 7.0 was selected as the working electrolyte pH. Also, pH 7.0 mimics biological conditions and thus chosen as the supporting electrolyte pH for all successive experiments.

**Figure 7:**
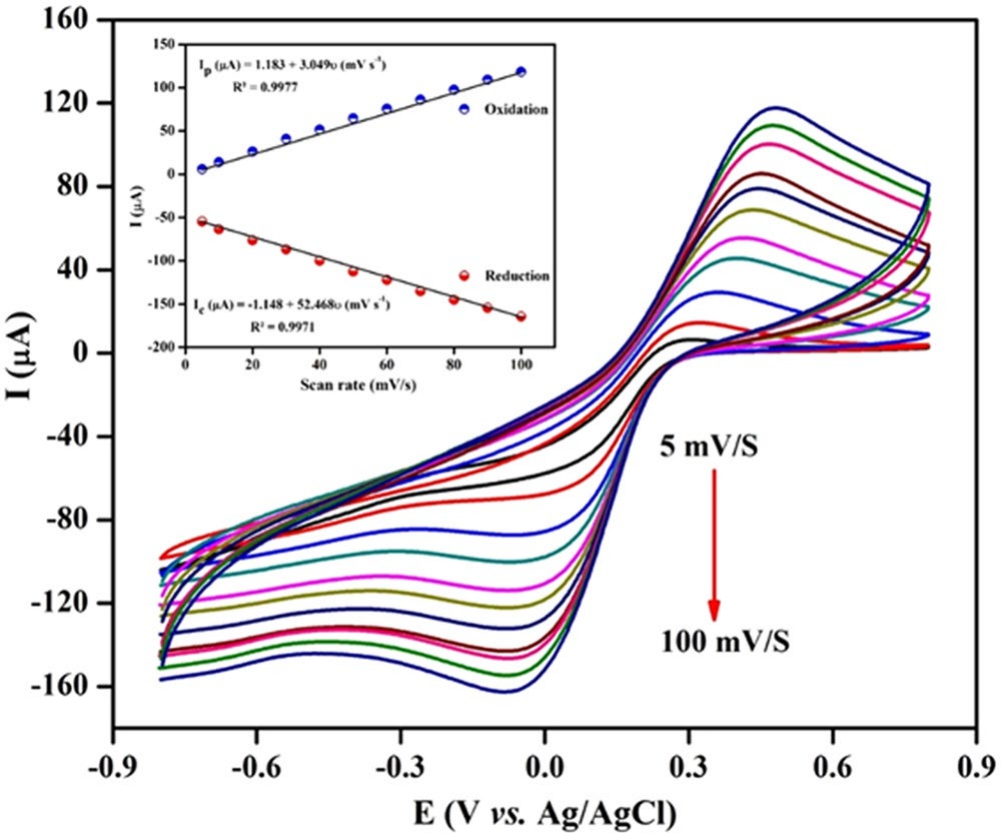
CVs response obtained for BSA/Anti-C_mab_/Fe_2_O_3_/CCY electrode as a function of scan rates (5 to 100 mV/s) in PBS (10 mM, pH@7) [Insert: Linear plot of the oxidation and reduction peak currents vs. scan rates].

The electrochemical behaviour of the BSA/Anti-C_mab_/Fe_2_O_3_/CCY immunoelectrode was also studied using CV as a function of scan rates from 5–100 mV/s in PBS (10 mM, pH 7.0) which is shown in Fig. 7 and the change in anodic and cathodic current response with scan rate is plotted in Fig. 7 as insert. It is observed that the magnitude of the current is linearly dependent on the scan rates and the equations are given in Eqs 1 and 2.

1 $${{\rm{I}}}_{{\rm{pa}}}({\rm{\mu }}{\rm{A}})=1.183{\rm{x}}+3.049{\rm{\upsilon }}({{\rm{mVs}}}^{-1});\,{{\rm{R}}}^{2}=0.9977\,$$

2 $${{\rm{I}}}_{{\rm{pc}}}({\rm{\mu }}{\rm{A}})=-\,1.148{\rm{x}}+52.468{\rm{\upsilon }}({{\rm{mVs}}}^{-1});\,{{\rm{R}}}^{2}=0.9971$$

The well-defined redox peak suggests that the diffusion of electron is surface controlled. The separation of peaks suggests that the process is not perfectly reversible but the stable redox peak current and position during the repeated scans at a particular scan rate suggests that the immunoelectrode exhibits a quasi-reversible process^7^. It is observed that the prepared immunoelectrode showed characteristic redox peak for Fe_2_O_3_ in PBS and exhibit stability up to 100 mV/s with slight peak – to – peak separation (ΔE_p_) of 122 mV, which evidenced the two electron transfer process. Additionally, the anodic peak to cathodic peak current ratio of (I_pc_/I_pa_) of prepared BSA/Anti-C_mab_/Fe_2_O_3_/CCY immunoelectrode is 0.822^52^. The obtained low working potential helps to avoid possible interference from the biological samples. Further, the fabricated BSA/Anti-C_mab_/Fe_2_O_3_/CCY immunoelectrode can be used as a mediator free electrochemical biosensor. It was found that at a scan rate of 50 mV/s, the electrode exhibit stability and equal oxidation and reduction peak area and current values. Thus, all further CV studies were carried out at a scan rate of 50 mV/s.

### Cortisol response studies of BSA/Anti-C_mab_/Fe_2_O_3_/CCY immunoelectrode

**Figure 8:**
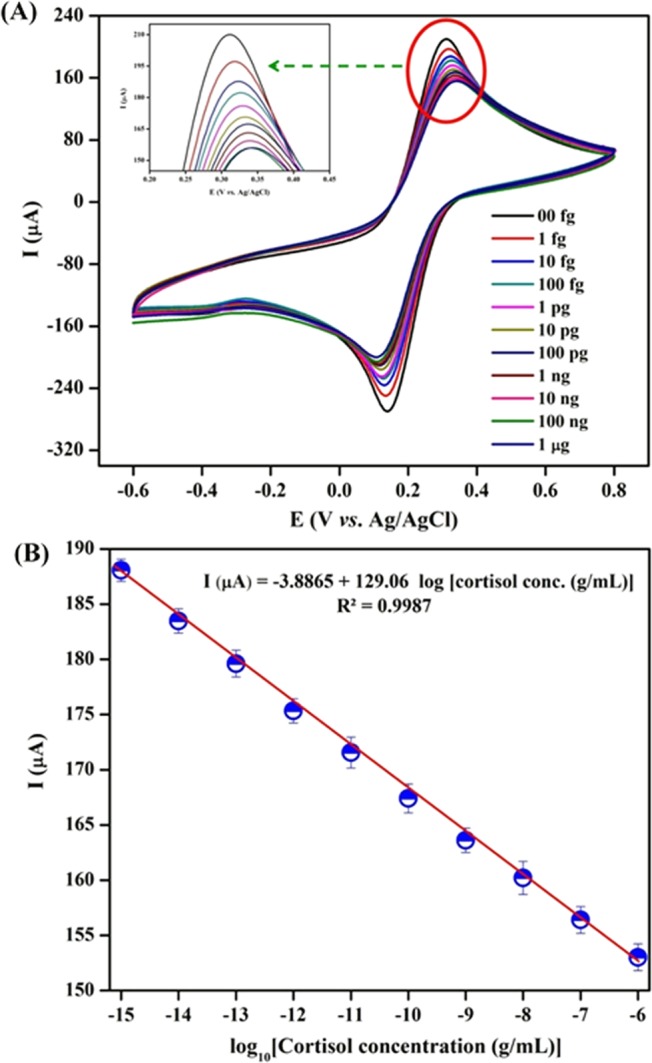
(**A**) Electrochemical response studies of BSA/Anti-C_mab_/Fe_2_O_3_/CCY immunoelectrode as a function of cortisol concentration varying from 1 fg to 1 μg in PBS (10 mM, pH 7.0); (**B**) Linear plot between electrochemical peak current response and logarithm of cortisol concentration.

The electrochemical response of BSA/Anti-C_mab_/Fe_2_O_3_/CCY immunoelectrode has been studied using CV technique using PBS (pH 7.0, 10 mM) at scan rate of 50 mV/s as a function of cortisol concentration ranging from 1 fg to 1 μg in three electrode system. Different BSA/Anti-C_mab_/Fe_2_O_3_/CCY immunoelectrode prepared in the same batch were found to exhibit similar current values with maximum variation of ±3%. The magnitude of the electrochemical current response decreases as a function of increasing cortisol concentration. The formation of insulating immune complex between Anti-C_mab_ and cortisol that hinders electron transport is attributed to decreased current response. This insulating behavior of cortisol binding was investigated and shown in Fig. 8A. As shown in Fig. 8B, the calibration curve between the current response and logarithm of cortisol concentration has been plotted in the range of 1 fg to 1 μg under optimized parameters. The linear regression equation was obtained as ΔI (μA) = −3.886 log [cortisol conc. (M)]. +129.06 with a correlation coefficient (R^2^) of 0.9987. The detection limit of the fabricated BSA/Anti-C_mab_/Fe_2_O_3_/CCY immunosensor is estimated as 0.005 fg using $3{{\rm{\sigma }}}_{{\rm{b}}}/{\rm{m}}$, ${{\rm{\sigma }}}_{{\rm{b}}}$ is the standard deviation and m is the slope. The results from three successive experiments (n = 3) are indicated by the error bars. The obtained detection limit of the fabricated immunosensors is lower when compared with other immunosensor reported in literature (Table 1). Therefore, the demonstrated method exhibited a good analytical performance for cortisol detection and could be used as a wearable platform for the detection of cortisol in real samples.

**Table 1:** Comparative analytical performance of BSA/Anti-C_mab_/Fe_2_O_3_/CCY electrode with other electrodes for the detection of cortisol.

Sensing Platform	Sensing molecule	Analyte	Transducers	Linear range	Detection limit	Ref.
Ag@AgO/PANI	Anti-C_mab_	Cortisol	CV	1 pM – 1 μM	0.64 pM	^7^
ZnO thinfilm on nanoporous polyamide membrane	Anti-C_mab_	Cortisol	EIS	1 pg – 100 ng	1 pg	^12^
HRP-Strept-Biotin-Ag-Cor/AuNPs/MrGO/Nafion@GCE	Anti-C_mab_	Cortisol	DPV	0.1–1000 ng/mL	0.05 ng	^23^
Antibody/d-BSA/rGO	Anti-C_mab_	Cortisol	EIS	10 pM- 100 nM	–	^26^
Au/PANI nanocomposite	Anti-C_mab_	Cortisol	CV	1 pM – 100 nM	1 pM	^41^
CNTs	Anti-C_mab_	Cortisol	Chemisresitive	1 pg – 10 ng	1 pg	^55^
Anti-C_ab_/ZnO-NFs/Au	Anti-C_mab_	Cortisol	CV	1 pM – 100 nM	1 pM	^56^
Magnetic nanoparticle/Screen printed carbon electrode	Anti-C_mab_	Cortisol	DPV	0.005–150 ng	3.5 pg	^57^
Protein G/DTBP scaffold/Au electrode	Anti-C_mab_	Cortisol	SWV	0.14–7 nM	16 pg	^58^
BSA/Anti-C_mab_/Fe_2_O_3_/CCY	Anti-C_mab_	Cortisol	CV	1 fg – 1 μg	0.005 fg	Present work

### Performance of BSA/Anti-C_mab_/Fe_2_O_3_/CCY immunoelectrode by DPV

**Figure 9:**
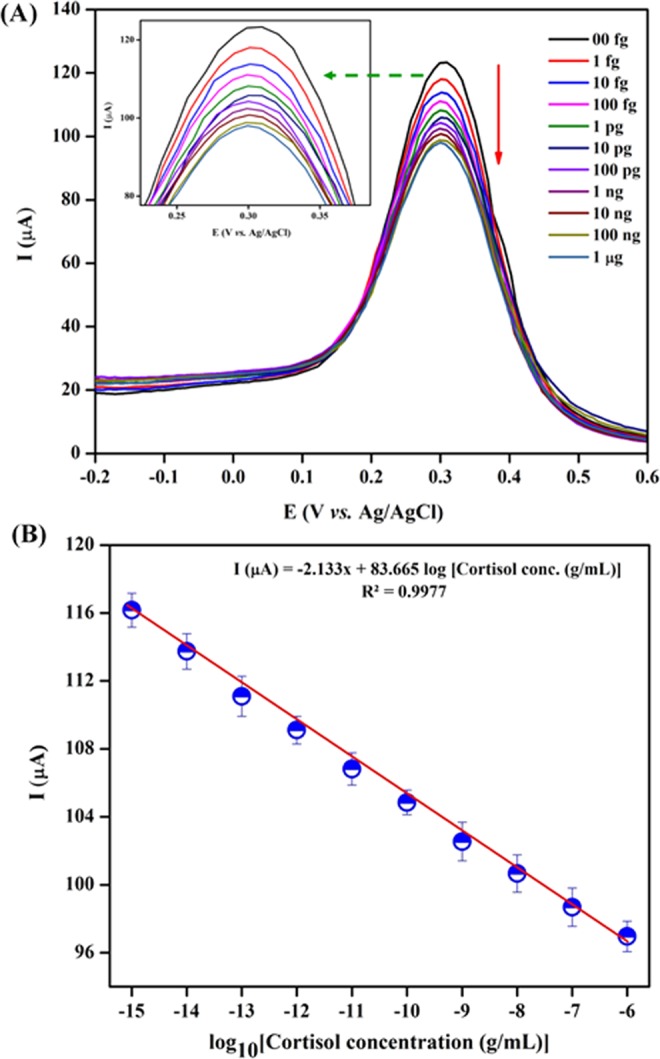
(**A**) DPV studies of the BSA/Anti-C_mab_/Fe_2_O_3_/CCY immunoelectrode as a function of cortisol concentration varying from 1 fg to 1 μg in PBS (10 mM, pH 7.0); (**B**) Linear plot between electrochemical peak current response and logarithm of cortisol concentration.

Also, the electrochemical response of BSA/Anti-C_mab_/Fe_2_O_3_/CCY immunoelectrode has been studied by DPV under similar condition used for CV. The DPV is sensitive analytical technique to study the electrochemical changes during biological reaction on the surface when the analyte concentration is very low^53^. Figure 9 revealed the peak current of the BSA/Anti-C_mab_/Fe_2_O_3_/CCY immunoelectrode decreasing when increasing the cortisol concentration. This confirms the effective formation of an immune-complex between antigen and antibody and the hindrance in electron transfer to the electrode due to the insulating behavior of cortisol. It is clear from Fig. 9A that linear curve attained between the logarithmic concentration of cortisol and decrement of current revealed good linear range from 1 fg – 1 μg. The linear regression equation was obtained as I (μA) = 2.133 + 83.665 log C_cor_ (g/mL) with a correlation coefficient (R^2^) of 0.9977. The detection limit was estimated to be about 0.003 fg/mL. The results from three successive experiments (n = 3) for the concentration on different immunoelectrode are indicted by the error bars. As the DPV method is highly sensitive compared to CV in trace level analyte detection, the lower detection of limit was calculated from DPV outcomes as 0.003 fg.

The high sensitivity of the BSA/Anti-C_mab_/Fe_2_O_3_/CCY immunoelectrode might resulted from high specific surface area and electrochemically accessible active surface area. The high specific surface area of Fe_2_O_3_/CCY (146.02 m^2^/g) is calculated using BET analysis for ellipsoid morphological Fe_2_O_3_ which is higher than the other morphologies given in the previous reports. Also, the electrochemically accessible active surface area (A_e_) were calculated using the standard Randle–Sevcik equation. The calculated A_e_ values (bare CCY is 0.0700 cm^2^ and Fe_2_O_3_/CCY is 0.0802 cm^2^) also ensured the high sensitivity of the prepared electrode^54^.

### Interference studies

**Figure 10:**
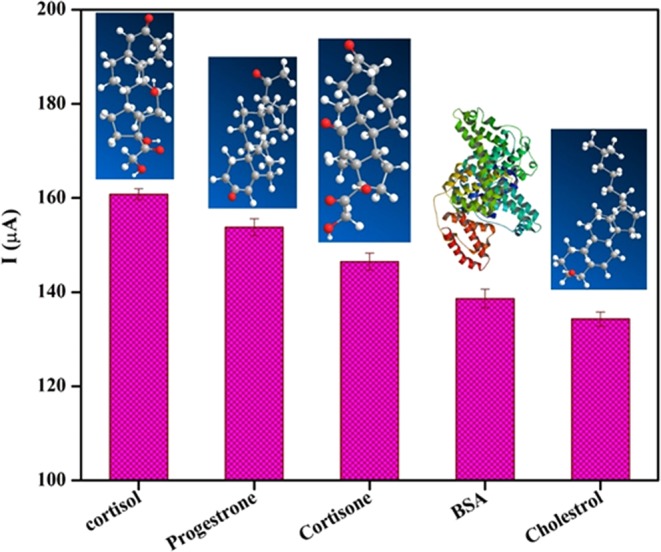
Interference studies of BSA/Anti-C_mab_/Fe_2_O_3_/CCY immunoelectrode using progesterone (100 fg/mL), cortisone (100 fg/mL), BSA (100 fg/mL) and cholesterol (100 fg/mL) with respect to cortisol (100 fg/mL) in PBS (10 mM, pH 7.0).

It is also important to evaluate how specifically and precisely the proposed sensing platform can detect cortisol in the presence of various interfering samples. The selectivity of the BSA/Anti-C_mab_/Fe_2_O_3_/CCY immunoelectrode towards cortisol (100 fg/mL) have been tested with interference especially cortisol analogous, for progesterone (100 fg/mL), cortisone (100 fg/mL), BSA (100 fg/mL) and cholesterol (100 fg/mL) using CV technique in PBS (10 mM, pH 7.0). As shown in Fig. 10, CV spectra exhibited a clear distinction of cortisol over progesterone, cortisone, BSA and cholesterol and decrement of electrochemical current response upto 8% to cortisol analogous, it can be concluded that the flexible and mediator free immunosensor electrode is highly selective towards cortisol estimation.

### Reproducibility and stability of the immunosensor

To examine the repeatability of the BSA/Anti-C_mab_/Fe_2_O_3_/CCY immunoelectrode, five separate electrodes were prepared and analyzed by CV technique. The average relative standard deviation (RSD) of immunosensor was found to be 3.48% for five measurements of 100 fg/mL of cortisol. Additionally, the immunosensor was stored at 4 °C for 30 days and it was used to detect cortisol samples. The BSA/Anti-C_mab_/Fe_2_O_3_/CCY immunosensor still retained 95.28% of its response. The excellent stability of the immunosensor attributed to the strong interaction to the cortisol. The slight decrement in response might be due to the long-term deactivation of the immobilized biomolecules. Therefore, these results specified the fabricated immunosensor has agreeable reproducibility and stability.

### Determination of cortisol in sweat samples

**Figure 11:**
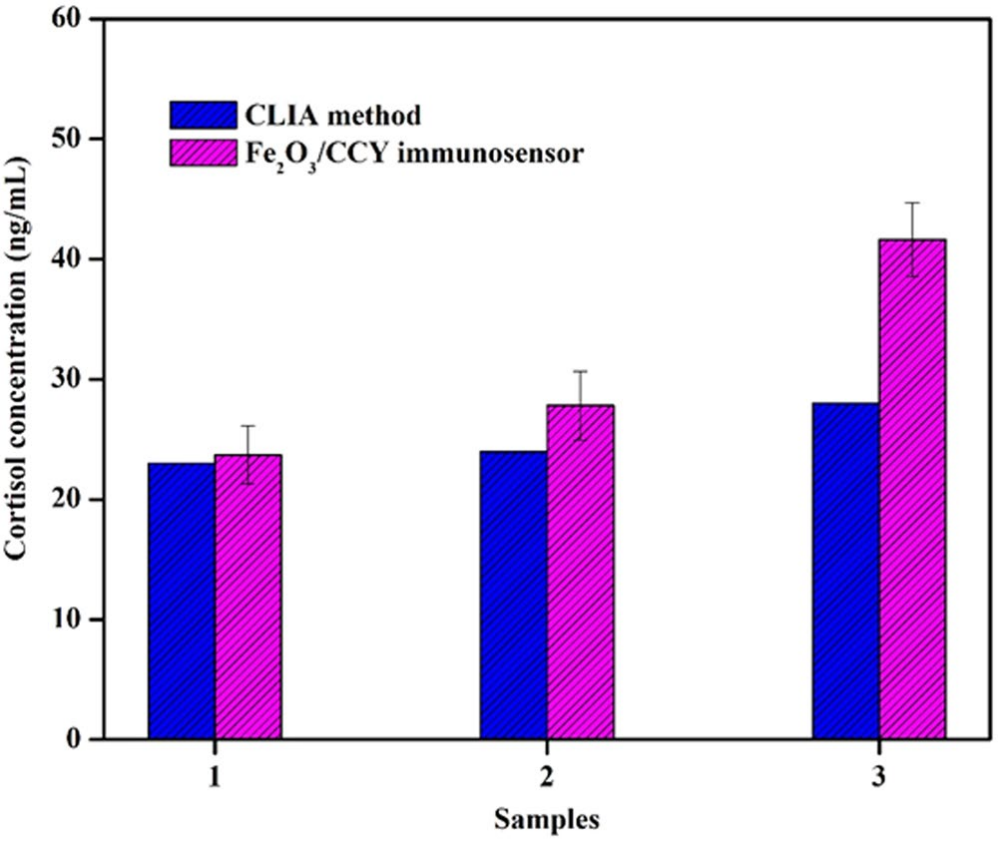
Comparison graph of sweat cortisol estimated using chemiluminescence immunoassay and Fe_2_O_3_/CCY based cortisol immunosensor.

We further examined the practicability of prepared immunosensor through analyzing real sweat samples and the results are given in Table 2. Herein, CV method was used to detect the cortisol level in sweat. The RSD of the proposed immunosensor from 3.403% to 4.064% and the recovery rates of the samples ranged between 99.62% and 104.21%. The significant recovery percentage of cortisol in various sweat samples were determined. The outcome values were validated using commercially available CLIA sensing method as shown in Fig. 11. The sweat cortisol readings were validated using commercial chemiluminescence immunoassay (CLIA) kit purchased from Abbott Diagnostics (IL, USA). This is a competitive CLIA which uses polyclonal anticortisol antibodies (Supplementary Materials ESI: S2). A good correlation between the electrochemical measurements and CLIA results was observed. The results of both techniques are summarized and tabulated (Table 2).

**Table 2:** Comparison of sweat cortisol estimated using chemiluminescence immunoassay and Fe_2_O_3_/CCY based cortisol immunosensor.

Samples	Chemiluminescence immunoassay (CLIA) method (ng/mL)	Fe_2_O_3_/CCY immunosensor				
		Measured (ng/mL)*	Added (ng/mL)	Found (ng/mL)*	RSD (%)	Recovery (%)
1	23	23.71	50	75.24	3.403	102.07
2	24	27.81	50	77.51	3.874	99.62
3	28	41.62	50	95.48	4.064	104.21

*The average value of three successive experiments.

## Conclusions

A highly sensitive and conductive yarn based flexible electrochemical sensor has been developed for the cortisol detection in sweat. EDC/NHS chemistry has been used to immobilize specific cortisol antibodies on the electrode surface. The immunosensor electrode successfully exhibits a sensing range of 1 fg – 1g, detection limit of 0.005 fg/mL with the regression coefficient of 0.9987. The immunoelectrode indicated excellent selectivity to cortisol when compared to cortisol analogous such as cortisone and progesterone and cholesterol. Cortisol detection recovery was 99.62 to 104.21% in human sweat samples. Response time of the immunosensor is 120 sec and the sensing results are correlated well with chemiluminescence immunoassay. The proposed conductive yarn based system has great potential for commercialization as this platform could be readily integrated with fabrics and garments.

## Supplementary information

Supplementary Information

## References

[1] Qureshi A, Gurbuz Y, Niazi JH (2012). Biosensors for cardiac biomarkers detection: A review. Sens. Actuator B-Chem..

[2] Martin-Ventura JL (2009). Biomarkers in cardiovascular medicine. Rev Esp Cardiol..

[3] Bairey MCN (2002). Psychosocial stress and cardiovascular disease: pathophysiological links. Behav Med..

[4] Anastasova S (2017). wearable multisensing patch for continuous sweat monitoring. Biosens. Bioelectron..

[5] Kamei T (1998). Physical stimuli and emotional stress-induced sweat secretions in the human palm and forehead. Anal. Chim. Acta.

[6] Venugopal M, Arya SK, Chornokur G, Bhansali S (2011). A Real time and Continuous Assessment of Cortisol in ISF Using Electrochemical Impedance Spectroscopy. Sens Actuators A - Phys..

[7] Kaushik A, Vasudev A, Arya SK, Bhansali S (2013). Mediator and label free estimation of stress biomarker using electrophoretically deposited Ag at AgO-polyaniline hybrid nanocomposite. Biosens. Bioelectron..

[8] Lupien SJ, McEwen BS, Gunnar MR, Heim C (2009). Effects of stress throughout the lifespan on the brain, behaviour and cognition. Nat. Rev. Neurosci..

[9] Arya SK, Chornokur G, Venugopal M, Bhansali S (2010). Dithiobis(succinimidyl propionate) modified gold microarray electrode based electrochemical immunosensor for ultrasensitive detection of cortisol. Biosens. Bioelectron..

[10] Djuric Z (2008). Biomarkers of Psychological Stress in Health Disparities Research. Open Biomark J..

[11] Kaushik A, Vasudev A, Arya SK, Pasha SK, Bhansali S (2014). Recent advances in cortisol sensing technologies for point-of-care application. Biosens. Bioelectron..

[12] Munje RD, Muthukumar S, PanneerSelvam A, Prasad A (2015). Flexible nanoporous tunable electrical double layer biosensors for sweat diagnostics. Sci. Rep..

[13] Appel D, Schmid RD, Dragan CA, Bureik M, Urlacher VB (2005). A fluorimetric assay for cortisol. Anal. Bioanal. Chem..

[14] Oka K, Noguchl M, Kltamura T, Shims S (1987). Liquid Chromatography and Radio immunoassay Compared for Determination of Cortisol and Corticosterone in Plasma after a Dexamethasone Suppression Test. Clin. Chem..

[15] Gatti R (2005). Urinary high performance reverse phase chromatography cortisol and cortisone analyses before and at the end of a race in elite cyclists. J. Chromatogr. B.

[16] Sarkar M (2007). Application of sensitive enzyme immunoassay for determination of cortisol in blood plasma of yaks (Poephagusgrunniens L.). Gen. Comp. Endocrinol..

[17] Shimada M, Takahashi K, Ohkawa T, Segawa M, Higurashi M (1995). Determination of salivary cortisol by ELISA and its application to the assessment of the circadian rhythm in children. Horm. Res..

[18] Schmalzing D (1995). Capillary Electrophoresis-Based Immunoassay for Cortisol in Serum. Anal. Chem..

[19] Zhou JC (2004). Immunoassays for cortisol using antibody-doped sol–gel silica. J. Mater. Chem..

[20] Stevens RC, Soelberg SD, Near S, Furlong CF (2008). Detection of Cortisol in Saliva with a Flow-Filtered, Portable Surface Plasmon Resonance Biosensor System. Anal. Chem..

[21] Fernandez RE (2017). Disposable aptamer-sensor aided by magnetic nanoparticle enrichment for detection of salivary cortisol variations in obstructive sleep apnea patients. Sci. Rep..

[22] Manickam P, Pasha, Shedra SK, Snipes A, Bhansali S (2017). A Reusable Electrochemical Biosensor for Monitoring of Small Molecules (Cortisol) Using Molecularly Imprinted Polymers. J. Electrochem. Soc..

[23] Sun B (2017). Investigate electrochemical immunosensor of cortisol based on gold nanoparticles/magnetic functionalized reduced graphene oxide. Biosens. Bioelectron..

[24] Li F (2016). An ultrasensitive label-free electrochemical immunosensor based on signal amplification strategy of multifunctional magnetic graphene loaded with cadmium ions. Sci. Rep..

[25] Dey A, Kaushik A, Arya AK, Bhansali S (2012). Mediator free highly sensitive polyaniline–gold hybrid nanocomposite based immunosensor for prostate-specific antigen (PSA) detection. J. Mater. Chem..

[26] Kim KS (2017). Highly sensitive and selective electrochemical cortisol sensor using bifunctional protein interlayer-modified grapheme electrodes. Sens. Actuator B-Chem..

[27] Gao W (2016). Fully integrated wearable sensor arrays for multiplexed *in situ* perspiration analysis. Nature.

[28] Bai J, Qi P, Ding X, Zhang H (2016). Graphene composite coated carbon fiber: electrochemical synthesis and application in electrochemical sensing. RSC Adv..

[29] Liu Y (2015). SnO_2_ coated carbon cloth with surface modification as Na-ion battery anode. Nano Energy.

[30] Chand S (2000). Carbon fibers for composites. J. Mater. Sci..

[31] Madhu S (2018). Nanostructured SnO_2_ integrated conductive fabrics as binder-free electrode for neurotransmitter detection. Sens. Actuators, A.

[32] Shim BS, Chen W, Doty C, Xu C, Kotov NA (2008). Smart electronic yarns and wearable fabrics for human biomonitoring made by carbon nanotube coating with polyelectrolytes. Nano Lett..

[33] Pasche S (2008). Wearable Biosensors for Monitoring Wound Healing. Adv. Sci. Technol..

[34] Xu Y (2011). Uniform hematite α-Fe_2_O_3_ nanoparticles: Morphology, size-controlled hydrothermal synthesis and formation mechanism. Mater. Lett..

[35] Sun P (2012). Template-free synthesis of monodisperse α-Fe_2_O_3_ porous ellipsoids and their application to gas sensors. CrystEngComm..

[36] Mahesh KPO, Shown I, Chen LC, Chen KH, Tai Y (2018). Flexible sensor for dopamine detection fabricated by the direct growth of α-Fe_2_O_3_ nanoparticles on carbon cloth. Appl. Surf. Sci..

[37] Kwak YH, Kim W, Park KB, Kim K, Seo S (2017). Flexible heartbeat sensor for wearable device. Biosens. Bioelectron..

[38] Wu S, Liu P, Zhang Y, Zhanga H, Qin X (2017). Flexible and conductive nanofiber-structured single yarn sensor for smart wearable devices. Sens. Actuator B-Chem..

[39] Schazmann B (2010). A wearable electrochemical sensor for the real-time measurement of sweat sodium concentration. Anal. Methods.

[40] Manickam P, Sekar M, Fernandeza R, Viswanathan C, Bhansali S (2017). Fabric Based Wearable Biosensor for Continuous Monitoring of Steroids. ECS Trans..

[41] Arya SK, Dey A, Bhansali S (2011). Polyaniline protected gold nanoparticles based mediator free electrochemical cortisol biosensor and label free electrochemical cortisol biosensor. Biosens. Bioelectron..

[42] Russell E, Koren G, Rieder M, Uum SV (2014). The detection of cortisol in human sweat: implications for measurement of cortisol in hair. Ther Drug Monit..

[43] Sun C, Chen S, Li Z (2018). Controllable synthesis of Fe_2_O_3_-carbon fiber composites via a facile sol-gel route as anode materials for lithium ion batteries. Appl. Surf. Sci..

[44] Wang X (2016). Three-dimensional core-shell Fe_2_O_3_@carbon/carbon cloth as binder-free anode for the high-performance lithium-ion batteries. Appl. Surf. Sci..

[45] Song J (2015). Combination of nitrogen plasma modification and waterborne polyurethane treatment of carbon fiber paper used for electric heating of wood floors. Bioresources.

[46] Zou Y, Kan J, Wang Y (2011). Fe_2_O_3_-Graphene Rice-on-Sheet Nanocomposite for high and fast lithium ion storage. J. Phys. Chem. C.

[47] Qiu Q (2011). Microwave-Assisted Hydrothermal Synthesis of Nanosized α-Fe_2_O_3_ for Catalysts and Adsorbents. J. Phys. Chem. C.

[48] Ma HF (2015). Electrochemical determination of dopamine using octahedral SnO_2_ nanocrystals bound to reduced graphene oxide nanosheets. Microchim. Acta.

[49] Manivel P (2013). Conducting polyaniline-graphene oxide fibrous nanocomposites: preparation, characterization and simultaneous electrochemical detection of ascorbic acid, dopamine and uric acid. RSC Adv..

[50] Jian Z (2014). Fe_2_O_3_ nanocrystals anchored onto graphene nanosheets as the anode material for low-cost sodium-ion batteries. Chem. Commun..

[51] Chen LF, Yu ZY, Ma X, Li ZY, Yu SH (2014). *In situ* hydrothermal growth of ferric oxides on carbon cloth for low-cost and scalable high-energy-density supercapacitors. Nano Energy.

[52] Kokulnathan T, Anthuvan AJ, Chen MC, Chinnuswamy C, Kadirvelu K (2018). Trace level electrochemical determination of the neurotransmitter dopamine in biological samples based on iron oxide nanoparticle decorated graphene sheets. Inorg. Chem. Front..

[53] Sriramprabha R, Divagar M, Mangalaraj D, Ponpandian N, Viswanathan C (2015). Formulation of SnO_2_/graphene nanocomposite modified electrode for synergetic electrochemical detection of dopamine. Adv. Mater. Lett..

[54] Xu WH (2014). Investigation of facet-dependent performance of α-Fe_2_O_3_ nanocrystals for heavy metals determination by stripping voltammetry. Chem. Commun..

[55] Tlili C, Myung NV, Shetty V, Mulchandani A (2011). Label-free, chemiresistor immunosensor for stress biomarker cortisol in saliva. Biosens. Bioelectron..

[56] Vabbina PK, Kaushik A, Pokhrel N, Bhansali S, Pala N (2015). Electrochemical cortisol immunosensors based on sonochemically synthesized zinc oxide 1D nanorods and 2D nanoflakes. Biosens. Bioelectron..

[57] Moreno-Guzmán M (2010). Disposable immunosensor for cortisol using functionalized magnetic particles. Analyst.

[58] Liu X (2011). Detection of cortisol at a gold nanoparticle|Protein G–DTBP-scaffold modified electrochemical immunosensor. Analyst.

