# Supplementary material for: Carbon fiber based electrochemical sensor for sweat cortisol measurement
Source: Sci Rep. 2019 Jan 23;9:403. doi: 10.1038/s41598-018-37243-w (PMC6344552; doi:10.1038/s41598-018-37243-w)
Supplement: Supplementary file 1 — Supplementary Information [file 41598_2018_37243_MOESM1_ESM.docx]

**Supporting material**

**Carbon fiber based electrochemical sensor for sweat cortisol measurement**

**Sekar Madhu^1^, Pandiaraj Manickam^2^, Shekhar Bhansali ^3^, N. Ponpandian^1^**

**& Viswanathan Chinnuswamy^1^***

*^1^ Department of Nanoscience & Technology, Bharathiar University, Coimbatore 641 046, India.*

*^2^ Electrodics and Electrocatalysis Division, CSIR-Central Electrochemical Research Institute, Karaikudi-630006, Tamil Nadu, India.*

*^3^ Bio-MEMS and Microsystems Laboratory, Department of Electrical and Computer Engineering, Florida International University, Miami, FL, USA.*

*Corresponding author. Tel: (+91) 422-2428422, Fax: (+91) 422-2422387
*E-mail address*: viswanathan@buc.edu.in (C. Viswanathan)

**S1. Stability of biosensor**

The stability of CCY and Fe_2_O_3_/CCY (Hanged up straight and bend) was investigated by measuring the current response using cyclic voltammetry for 500 cycles in 0.1 M PBS. The relative standard deviation (RSD) values were calculated from the current responses obtained for Fe_2_O_3_/CCY hanged up straight (Figure S1b) and bend (Figure S1c) are 1.365% and 1.184% respectively. The initial values and after 500 rounds of cyclic scanning in straight and bending movement are almost similar, indicating the good stability the fiber. Thus, the bending or stretching movement of the modified electrode has a very margin influence on the electrochemical performance of the sensor system. The Fe_2_O_3_ integrated CCY is electrochemically stable for these biosensor applications.


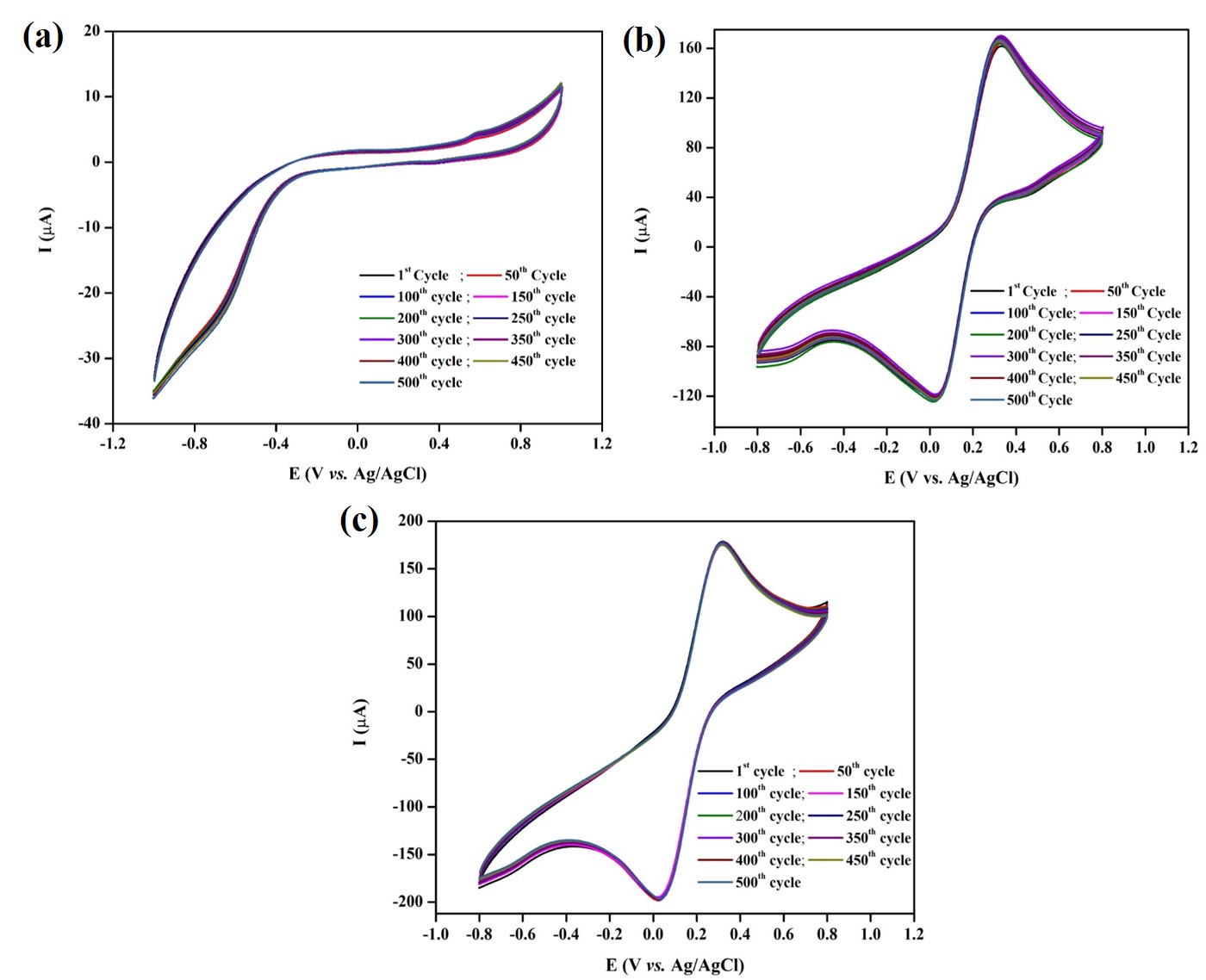


**Figure S1.** The stability tests of (a) bare; (b) Fe_2_O_3_/CCY (Hanged up straight); (c) Fe_2_O_3_/CCY (Hanged up bend) for 500 cycles in the 0.1 M PBS at scan rate of 50 mV/s.

**S2. Determination of cortisol in real sample**

We examined the practicability of prepared immunosensor through analyzing real sweat samples and the results are given in Table 2. Herein, CV method was used to detect the cortisol level in sweat. The RSD of the proposed immunosensor from 3.403% to 4.064% and the recovery rates of the samples ranged between 99.62% and 104.21%. The significant recovery percentage of cortisol in various sweat samples were determined. The outcome values were validated using commercially available CLIA sensing method.

CLIA is one of the widely used methods, currently being used in the laboratories to measure biological levels of hormones, drugs, vitamins, tumor markers, infectious disease markers, myocardial damage markers and autoantibodies. The sweat cortisol readings were validated using commercial chemiluminescence immunoassay (CLIA) kit purchased from Abbott Diagnostics (IL, USA). This is a competitive CLIA which uses polyclonal anticortisol antibodies.

Recommended assay protocols were followed to analyse salivary cortisol levels. In brief, sample and anti-cortisol coated magnetic microparticles are combined to create a reaction mixture. Cortisol present in the sample binds to the anti-cortisol coated microparticles. After incubation, cortisol acridinium-labeled conjugate is added to the reaction mixture. The cortisol acridinium-labeled conjugate competes for the available binding sites on the anti-cortisol coated microparticles. Following a second incubation, the microparticles are washed, and pre-trigger and trigger solutions are added to the reaction mixture. The resulting chemiluminescent reaction is measured as relative light units (RLUs). A good correlation between the electrochemical measurements and CLIA results was observed. The results of both techniques are summarized and tabulated below,

**Table S1.** Comparison of sweat cortisol estimated using chemiluminescence immunoassay and Fe_2_O_3_/CCY based cortisol immunosensor.

| **Samples** | **Chemiluminescence immunoassay (CLIA) method**  **(ng/mL)** | **Fe_2_O_3_/CCY immunosensor** | | | | |
| --- | --- | --- | --- | --- | --- | --- |
|  |  | **Measured**  **(ng/mL)*** | **Added**  **(ng/mL)** | **Found**  **(ng/mL)*** | **RSD**  **(%)** | **Recovery**  **(%)** |
| 1 | 23 | 23.71 | 50 | 75.24 | 3.403 | 102.07 |
| 2 | 24 | 27.81 | 50 | 77.51 | 3.874 | 99.62 |
| 3 | 28 | 41.62 | 50 | 95.48 | 4.064 | 104.21 |

* The average value of three successive experiments.
